# Glasgow Prognostic Score as a predictive factor differentiating surgical site infection and remote infection following colorectal cancer surgery?

**DOI:** 10.1038/sj.bjc.6605353

**Published:** 2009-10-06

**Authors:** C Miki, Y Mohri, Y Toiyama, T Araki, K Tanaka, Y Inoue, K Uchida, M Kusunoki

**Affiliations:** 1 Department of Gastrointestinal and Paediatric Surgery, Mie University Graduate School of Medicine, Edobashi 2-174, Tsu, Mie 514-8507, Japan

**Sir,**

We read with interest the recent article indicating that a preoperative systemic inflammatory response is an underlying host characteristic that predisposes to postoperative infection in patients undergoing potentially curative resection for colorectal cancer. In this article, the Glasgow group found that elevated C-reactive protein together with low albumin levels (Glasgow prognostic score, GPS) were the only host-associated factors that had a high predictive value, which included 40% of patients affected by postoperative infectious complications (Moyes *et al*, 2009).

Postoperative infectious complications are usually classified into surgical site infections (SSIs) and remote infections. An SSI as represented by wound infection and anastomotic leak is an infection that occurs after surgery in the part of the body where the surgery occurred, and is further classified into incisional and organ/space SSIs. In contrast, a remote infection as represented by respiratory organ infection, infection via catheters, urinary tract infection, and antibiotic enterocolitis, is an exogenous and/or cross infection that occurs at sites not directly subjected to operations. With regard to aetiological bacteria of these infections, the onset of an SSI is usually caused by contaminants that exist in the operative fields. The onset of a remote infection is often caused by antibiotic-resistant bacteria that cause nosocomial infections, such as *Pseudomonas aeruginosa* or methicillin-resistant *Staphylococcus aureus*. Thus, SSIs and remote infections may have a distinct pathogenesis, being specific to the different underlying mechanisms, and may differ from each other with respect to their risk factors (Edwards, 1976). It is important to identify which clinical and laboratory factors can predict site-specific patterns of infectious complications in SSIs and remote infections, because it may assist surgeons with specific strategies for prevention of infection according to each patient's risk factor. However, the authors did not evaluate measurable preoperative clinical and laboratory markers that may be able to predict site-specific patterns of postoperative infectious complications in SSIs and remote infections.

In our previous study, we determined site-specific patterns of risk factors for incisional and organ/space SSIs in 285 colorectal cancer patients to develop predictive models using inflammatory mediators (Miki *et al*, 2006). We found that operating time and concomitant medical problems were independently associated with the development of incisional SSIs, whereas operating time, tumour location, obesity and concomitant medical problems were independently associated with the development of organ/space SSIs. However, we were not able to distinguish clinical factors that could predict remote infections from those that could predict SSIs because of a limited number of patients. We would like to ask the authors to show whether GPS can predict site-specific patterns of postoperative infectious complications in SSIs and remote infections. We believe that GPS could differentiate the odds of developing SSI from the odds of developing remote infection, because they could successfully sort out only one host-associated factor from a large number of patients (*n*=455) and the patients with postoperative complications could be nearly equally divided into an SSI group (*n*=31) and a remote infection group (*n*=39) If the odds of developing rates of SSI and remote infection are identified for each risk factor, then the GPS can be used by the surgeon to determine whether he/she should correct host-associated pathological states before surgery for preventing remote infection, or select less invasive surgery for minimising adverse effects of surgery-associated factors for preventing SSI. Although prospective randomised studies are required to clarify whether these suspected advantages can be realised in clinical practice, colorectal surgeons need to select from the strategies mentioned above for their cancer patients, if the GPS can differentiate the SSIs from remote infections.
